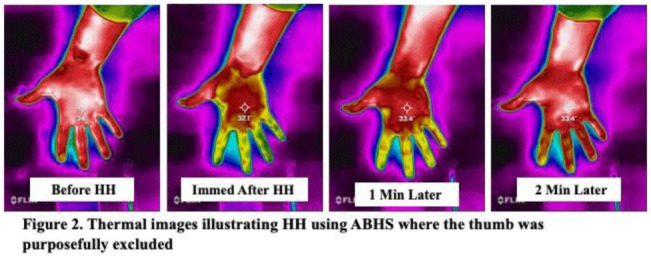# A pilot study of using thermal imaging to assess hand hygiene technique

**DOI:** 10.1017/ash.2022.148

**Published:** 2022-05-16

**Authors:** John Boyce, Richard Martinello

## Abstract

**Background:** Although substantial efforts have been made to improve hand hygiene (HH) compliance among healthcare personnel (HCP), much less attention has been devoted to improving HH technique. To date, no standard method for assessing HH technique has been widely adopted by hospitals. Because applying an alcohol-based hand sanitizer (ABHS) transiently reduces adjacent skin temperature, we explored the feasibility of using thermal imaging to determine whether ABHS has been applied to fingertips and thumbs, areas often missed by HCP. **Methods:** A convenience sample of 12 Quality and Safety staff volunteered for the study. A FLIR One Pro thermal camera attached to an iPhone was used to obtain thermal images of the palmar aspect of each volunteer’s dominant hand before applying ~1.8 mL ABHS gel, immediately after hands felt dry, and at 1 minute and 2 minutes later. Spot temperature readings of the mid-palm area and middle finger were recorded at each time point. The sex and estimated hand surface area (HSA in cm^2^) of each volunteer were recorded. **Results:** In 11 of 12 volunteers, thermal imaging showed a significant decrease in mid-palm and middle finger skin temperatures after performing HH (paired *t* test *P* < .01 for both), especially for the fingers and thumb, indicating that ABHS was applied to these areas (Fig. 1). When HH was performed with ABHS and the thumb was purposefully excluded, the lack of colorimetric change in the thumb was visible (Fig. 2). The palmar area showed the least drop in temperature and reverted to normal temperature more quickly. Immediate post-HH mid-palm temperature change ranged from +0.5 to −2.7°C, with a significantly greater mean temperature drop with small or medium hands than with large hands (Mann-Whitney *U* test *P* = .048). With some volunteers, the color changes lasted 1 minute or longer. However, for persons with “cold” fingers at baseline, it was more difficult to draw conclusions from the gross assessment for colorimetric change. **Conclusions:** Thermal imaging of HH performance shows promise as an HH assessment technique and may be useful to determine whether HCP have applied ABHS to their fingertips and thumbs. Additional studies involving a much larger number of HCP under varying conditions are needed to determine whether thermal imaging can be a practical modality for teaching HH technique, for routinely monitoring HH technique, or as a research tool for studying the dynamics of HH using ABHS.

**Funding:** None

**Disclosures:** None

**Figure:**
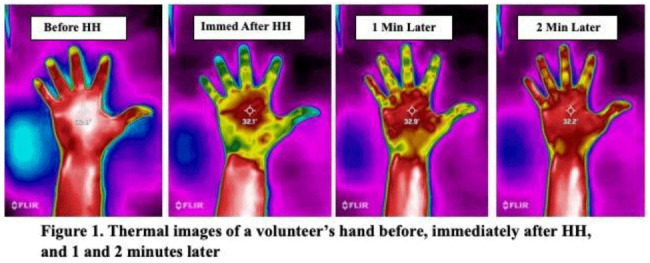

**Figure:**